# Metabolomic signatures of intestinal colonization resistance against *Campylobacter jejuni* in mice

**DOI:** 10.3389/fmicb.2023.1331114

**Published:** 2023-12-18

**Authors:** Nizar W. Shayya, Rasmus Bandick, Lia V. Busmann, Soraya Mousavi, Stefan Bereswill, Markus M. Heimesaat

**Affiliations:** Gastrointestinal Microbiology Research Group, Institute of Microbiology, Infectious Diseases and Immunology, Charité - University Medicine Berlin, Berlin, Germany

**Keywords:** *Campylobacter jejuni*, colonization resistance, gut microbiota, metabolome, bile acids, amino acids, fatty acids, host-pathogen interactions

## Abstract

**Introduction:**

*Campylobacter jejuni* stands out as one of the leading causes of bacterial enteritis. In contrast to humans, specific pathogen-free (SPF) laboratory mice display strict intestinal colonization resistance (CR) against *C. jejuni*, orchestrated by the specific murine intestinal microbiota, as shown by fecal microbiota transplantation (FMT) earlier.

**Methods:**

Murine infection models, comprising SPF, SAB, hma, and mma mice were employed. FMT and microbiota depletion were confirmed by culture and culture-independent analyses. Targeted metabolome analyses of fecal samples provided insights into the associated metabolomic signatures.

**Results:**

In comparison to hma mice, the murine intestinal microbiota of mma and SPF mice (with CR against *C. jejuni*) contained significantly elevated numbers of lactobacilli, and Mouse Intestinal Bacteroides, whereas numbers of enterobacteria, enterococci, and *Clostridium coccoides* group were reduced. Targeted metabolome analysis revealed that fecal samples from mice with CR contained increased levels of secondary bile acids and fatty acids with known antimicrobial activities, but reduced concentrations of amino acids essential for *C. jejuni* growth as compared to control animals without CR.

**Discussion:**

The findings highlight the role of microbiota-mediated nutrient competition and antibacterial activities of intestinal metabolites in driving murine CR against *C. jejuni*. The study underscores the complex dynamics of host-microbiota-pathogen interactions and sets the stage for further investigations into the mechanisms driving CR against enteric infections.

## 1 Introduction

In humans, the Gram-negative spirally curved *Campylobacter jejuni* is one of the common causative agents of bacterial enteritis (Igwaran and Okoh, 2019). Campylobacteriosis is initially characterized by severe enteritis with diarrhea and lower gastrointestinal tract hemorrhage. Less frequently, other non-gastrointestinal aftermaths are associated with campylobacteriosis such as systemic manifestations, which include infectious complications like bacteremia, and post-infectious immune disorders, like reactive arthritis or Guillain-Barré syndrome (Peterson, 1994; Heimesaat et al., 2021).

In the course of zoonotic infections *C. jejuni* is transmitted to humans via the oral route by consuming contaminated raw or undercooked meat, unpasteurized milk, in addition to uncooked cross-contaminated food, whereas human transmission is possible via the fecal-oral route (Kaakoush et al., 2015). To establish infection, *C. jejuni* colonizes the intestinal lumen, penetrates the mucus layer, adheres to epithelial cells, and invades subepithelial tissues. This is achieved via flagella, adhesins, and invasins (Heimesaat et al., 2014; Schmidt et al., 2019; Tegtmeyer et al., 2021). In the subepithelial tissues *C. jejuni* induces an inflammatory response by innate immune system activation via its surface endotoxin, the lipo-oligosaccharide (LOS) (Mousavi et al., 2020). The majority of strains causing disease lack conventional exotoxins, but cytolethal distending toxin or cholera-like toxins have been detected in some pathogenic strains and may increase symptom severity (Lara-Tejero and Galán, 2000).

The human host is highly susceptible to *C. jejuni* infection as emphasized by a low infection dosage (Igwaran and Okoh, 2019). In contrast to humans, conventional specific pathogen-free (SPF) laboratory mice are completely resistant to *C. jejuni* infection (Bereswill et al., 2011; Mousavi et al., 2021). Research in murine laboratory models revealed that the resistance to infection is caused by the inability of *C. jejuni* to multiply and to establish stable colonization in the presence of the specific intestinal microbiota present in conventional mice (Bereswill et al., 2011; Mousavi et al., 2021). This protective shield against bacterial infection is termed colonization resistance (CR) and is in general attributed to diverse antagonistic mechanisms including antimicrobial metabolites, nutrient depletion and/or bacteriophages (Lawley and Walker, 2013; Herzog et al., 2023). Notably, CR has gained considerable attention in research exploring the protective effects of intestinal microbiota against *Salmonella enterica* (Wotzka et al., 2019), *Escherichia coli* (Mundy et al., 2006), *Listeria monocytogenes* (Becattini et al., 2017), and *Clostridioides difficile* (Chen et al., 2008). Results from earlier studies demonstrated that CR against *C. jejuni* is caused by the specific murine microbiota (Bereswill et al., 2011). In contrast to SPF mice, secondary abiotic (SAB) mice in which the intestinal microbiota was completely depleted by antibiotic treatment display no CR and are highly susceptible to *C. jejuni* colonization (Bereswill et al., 2011; Mousavi et al., 2021; Heimesaat et al., 2022). Most importantly, CR was reconstituted in SAB mice upon recolonization with a murine intestinal microbiota, while SAB mice recolonized with a human intestinal microbiota lacked CR and were highly susceptible to *C. jejuni* colonization (Bereswill et al., 2011). Moreover, CR against *C. jejuni* was absent in mice with reduced species diversity of the intestinal microbiota, as both, antibiotics treated mice (O'Loughlin et al., 2015; Iizumi et al., 2016) and infant mice harboring a limited gut microbiota were proven liable to *C. jejuni* infection (Chang and Miller, 2006; Haag et al., 2012a; Stahl et al., 2014). Moreover, inflammation-induced dysbiosis shifts in the enteric microbiota composition toward elevated *E. coli* levels disrupted CR against *C. jejuni* as was observed independently in mice with *Toxoplasma gondii*-induced acute ileitis as well as in IL-10 deficient mice suffering from chronic colitis. Interestingly, intestinal *E. coli* loads were elevated in susceptible infant mice and CR against *C. jejuni* was abrogated by feeding of SPF mice with *E. coli* via the drinking water (Haag et al., 2012a,b).

However, molecular mechanisms involved in CR against *C. jejuni* have not been unraveled so far, but the causative role of the murine microbiota in CR indicates that nutrient competition and bactericidal killing by gut bacteria might play important roles therein. Unlike other enteric pathogens, *C. jejuni* generally lacks the ability to utilize carbohydrates as carbon sources and relies on organic acids and amino acids as primary nutrient sources (Hofreuter, 2014). Nevertheless, despite the restricted carbohydrate utilization, some fucose—catabolizing strains have been described such as *C. jejuni* NTC 11168 (Muraoka and Zhang, 2011).

Thus, amino acids are essentially required for *C. jejuni* multiplication and stable colonization in the gut (Hofreuter, 2014). Results from earlier investigations demonstrate that serine, aspartate, asparagine, and glutamate, and after that proline are used by the pathogen in a sequential manner (Wright et al., 2009). This rather limited array of substrates to fuel the central metabolism suggests that *C. jejuni* may effectively be outcompeted by other gut bacteria with similar substrate preferences. Additionally, the intestinal microbiota might provide CR by production of antimicrobials such as short-chain fatty acids (SCFAs), secondary bile acids or bacteriocins (Ducarmon et al., 2019). The role of SCFAs and bile acids in intestinal lifestyle of *C. jejuni* is evidenced by the fact that both substance groups are sensed by *C. jejuni* and regulate expression of factors involved in virulence and colonization (Ducarmon et al., 2019). Furthermore, *C. jejuni* is highly susceptible to killing by bile acids and has therefore, developed resistance mechanisms such as multidrug efflux pumps (Lin et al., 2003; Sun et al., 2018).

It seems feasible that the mechanisms involved in causing CR are key to eradicate enteric pathogens such as *C. jejuni* from the gut. Thus, better understanding the metabolic composition of the intestinal milieu in mice with and without CR will help to unravel the mechanisms underlying CR, and this might pave the way for developing novel drugs for prevention and treatment of campylobacteriosis.

To address these challenges and gain a comprehensive understanding of the mechanisms underlying murine CR against *C. jejuni*, we utilized a targeted metabolomics approach to characterize distinct metabolite patterns associated with CR in our well-established murine models of *C. jejuni* infection with known CR against the pathogen, namely, SPF mice, microbiota-depleted SAB mice, and SAB mice in which murine or human intestinal microbiota was reintroduced by fecal microbiota transplantation (FMT) via the oral route. In particular, the murine microbiota (mma) and human microbiota associated (hma) mice, both containing a complete intestinal microbiota of different composition, were used as study groups, whereas SAB and SPF mice served as controls.

## 2 Materials and methods

### 2.1 Mice

C57BL/6 J mice were maintained in the facilities of the “Forschunginstitute fur Experimentelle Medizin” (FEM, Charite – Universitätsmedizin, Berlin, Germany), under SPF conditions. Age and sex matched mice aged between 10 and 12 weeks were used.

### 2.2 Generation of SAB mice

To eradicate the commensal gut microbiota, mice were transferred to sterile cages and were subjected to an ampicillin plus sulbactam antibiotic regimen (2 g/L plus 1 g/L, respectively; purchased from Dr. Friedrich Ebert Arzneimittel, Ursensollen, Germany) as a drinking solution for a period of 8 weeks. These mice were kept and handled under strict aseptic conditions. Microbiota depletion was confirmed using cultural and molecular analyses of fecal samples, as described earlier (Heimesaat et al., 2022).

### 2.3 Fecal microbiota transplantation

Fresh fecal samples were collected from five individual healthy human volunteers and murine animals, respectively, after screening to ensure their exclusion of enteropathogenic bacteria, parasites, and viruses. Fecal samples were pooled separately for humans and mice and dissolved in an equal volume of sterile phosphate-buffered saline (PBS, Thermo Fischer Scientific, Waltham, MA, United States). Pooled samples were aliquoted and stored at −80°C until further use. Prior to gavage experiments, aliquots of the pooled fecal samples were thawed, and bacterial communities were analyzed using both culture-dependent and culture-independent methods with 16S rRNA gene sequencing. Quantification of total bacterial load and identification of individual bacterial groups were also done using cultural and culture-independent analyses. Starting a week prior to infection (i.e., on days −7, −6, and − 5), mice were gavaged with 300 μL of the respective fecal suspension as described in details earlier (Bereswill et al., 2011; Shayya et al., 2023), to study the effects of bacterial communities on host physiology and housed under SPF conditions with *ad libitum* access to food and water.

### 2.4 Gut microbiota analysis

Cultural analysis, biochemical identification, and molecular detection of luminal bacterial communities from stomach, duodenum, ileum, and colon were performed as previously described (Weschka et al., 2021; Shayya et al., 2023). In brief, DNA extracts and plasmids were quantified using Quat-iT PicoGreen reagent (Invitrogen, Paisley, UK) and adjusted to a concentration of 1 ng per μL. Abundance of the major bacterial groups within the gut microbiota was assessed using quantitative real time polymerase chain reaction (qRT-PCR) with group-specific primers targeting the 16S rRNA gene (Tib MolBiol, Berlin, Germany). The number of 16S rRNA gene copies per μg of DNA in each sample was determined, and the frequencies of the respective bacterial groups were computed relative to the eubacterial (V3) amplicon.

### 2.5 *Campylobacter jejuni* infection

For infection experiments, we utilized the *C. jejuni* strain 81-176, which was derived from frozen stocks and inoculated on karmali agar plates (Oxoid, Wesel, Germany). The bacteria were grown under microaerophilic conditions at 37°C in a closed container containing gas packs (CampyGen, Oxoid, Wesel, Germany) for 48 h to obtain optimal growth conditions. To establish an infection model, we infected SPF, SAB, mma, and hma mice with 10^9^ viable *C. jejuni* bacterial cells via oral gavage in a total volume of 300 μL of PBS on two consecutive days (days 0 and 1).

### 2.6 Sampling procedures

Mice were sacrificed by CO_2_ asphyxiation on day 21 post infection. Luminal samples from the gastrointestinal tract (stomach, duodenum, ileum, colon) of each mouse were harvested for a set of post-experimental analyses, including cultural and molecular analyses. Respective samples were homogenized in sterile PBS (Thermo Fisher Scientific, Waltham, MA, United States) and serial dilutions were plated onto karmali agar (Oxoid, Wesel, Germany) and incubated under microaerophilic conditions for 48 h at 37°C as described earlier in details (Heimesaat et al., 2022).

### 2.7 Metabolomic analysis

Targeted metabolomic analysis of fecal samples from d0 was performed by Biocrates Lifesciences AG (Innsbruck, Austria) using their McP Quant 500 platform. Fecal samples were collected from each subject and immediately frozen at −80°C until further analysis. Samples were shipped on dry ice to Biocrates for metabolomic analysis. Biocrates’ MxP^®^ Quant 500 product uses mass spectrometry (MS) to quantify over 500 metabolites in a single run, including amino acids, biogenic amines, organic acids, and lipids. The quality of the samples was assessed using internal standards, and data were preprocessed to remove contaminants, drift, and other systematic errors. Lipids and hexoses were analyzed using flow injection analysis-tandem mass spectrometry (FIA-MS/MS) on a SCIEX 5500 QTRAP^®^ instrument (AB SCIEX, Darmstadt, Germany) with an electrospray ionization (ESI) source. Small molecules were analyzed using liquid chromatography–tandem mass spectrometry (LC–MS/MS) on a Xevo^®^ TQ-XS instrument (Waters, Milford, MA, United States). To prepare the samples for analysis, a 96-well based sample preparation device was used, which contained inserts impregnated with internal standards. A predefined amount of the sample was added to the inserts, followed by the addition of a phenyl isothiocyanate (PITC) solution to derivatize some of the analytes (e.g., amino acids). After the derivatization was completed, the target analytes were extracted using an organic solvent, followed by a dilution step. The extracted metabolites were then analyzed by FIA-MS/MS and LC–MS/MS using multiple reaction monitoring (MRM) to detect the analytes. Concentrations of the metabolites were calculated using an appropriate mass spectrometry software (Sciex Analyst^®^ and Waters MassLynx^™^) and imported into Biocrates’ MetIDQ^™^ software for further analysis. The metabolite concentrations were then determined in each sample by normalizing to the weight and dilution factor from the original metabolome extraction protocol, then provided in “μM,” which were subsequently exported as an excel file. Individual metabolites were grouped by classes and visualized using GraphPad Prism (V9; San Diego, CA, United States).

### 2.8 Minimum inhibitory concentration determinations

The minimum inhibitory concentration (MIC) values for deoxycholic acid (Sigma-Aldrich, St. Louis, Missouri, United States; 30,960) were determined using the broth microdilution method in BactoTM Brain Heart Infusion (BHI) (BD Biosciences, Heidelberg, Germany) broth supplemented with 5% fetal bovine serum (FBS) (Biochrom, Berlin, Germany). The bacterial inoculum of *C. jejuni* 81-176 was prepared from overnight growth on karmali agar plates (Oxoid, Wesel, Germany) and suspended in 5 mL of BHI broth supplemented with 5% FBS to an optical density at 600 nm (OD600) of 0.1. Two-fold serial dilutions of the test compounds were prepared in 96-well plates in the range of 1–2,048 mg/L to a final volume of 200 μL per well, and 10 μL of the bacterial suspension was added to the respective wells. The plates were incubated for 48 h under microaerophilic conditions at 37°C, and then the OD was measured at 600 nm using a microplate reader (Infinite M Flex, Tecan, Switzerland). The MIC was defined as the lowest concentration of the compound that completely inhibited bacterial growth.

### 2.9 Statistical analysis

We conducted statistical analyses to evaluate the significance of observed differences between groups. Prior to analysis, we first assessed the normality of the data using the Anderson-Darling test. Medians and significance levels were then calculated using GraphPad Prism (V9; San Diego, CA, United States). For pairwise comparisons of the groups, we employed the Student’s *t*-test for normally distributed data and the Mann–Whitney test for non-normally distributed data. Moreover, to account for the possibility of errors resulting from multiple comparisons, we utilized the one-sided ANOVA with Tukey’s post correction for normally distributed data, and the Kruskal-Wallis test with Dunn’s post correction for non-normally distributed data. A two-sided probability (*p*) value of ≤0.05 was considered statistically significant.

### 2.10 Ethics statement

The animal studies were conducted in adherence to the European animal welfare guidelines (2010/64/EU) after receiving authorization from the local commission for animal experiments (“Landesamt für Gesundheit und Soziales.” LaGeSo, Berlin; under registration number G0172/16). Clinical conditions of mice after infection were surveyed daily.

## 3 Results

### 3.1 Survey of pathogen loads following *Campylobacter jejuni* infection

To establish CR against *C. jejuni* in SAB mice, and to confirm the role of the specific murine or human gut microbiota therein, we introduced human or murine gut microbiota to SAB mice by FMT via gavage. One week after reconstitution, these mice were perorally infected with 10^9^ viable *C. jejuni* on day (d) 0 and d1 along with conventional SPF mice and SAB mice as controls with and without CR, respectively. This setting enabled us to analyze CR by assessing the kinetics of pathogen shedding in fecal samples and in distinct gastrointestinal compartments.

**Figure 1:**
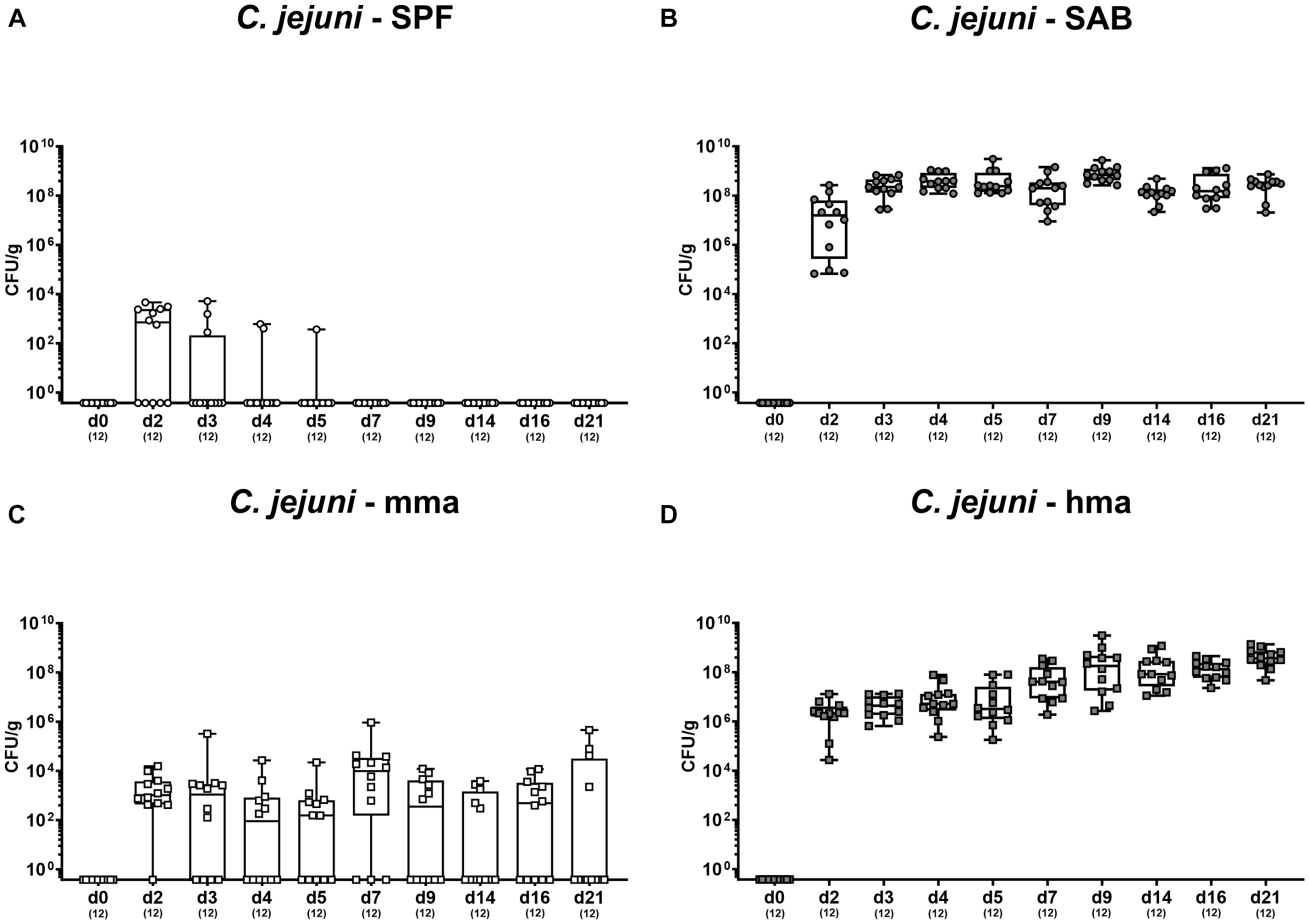
Survey of fecal pathogen loads over time post *C. jejuni* infection. Following peroral infection with *C. jejuni* strain 81-176 on day (d) 0 and d1, intestinal pathogen loads were surveyed in fecal samples over time post-infection from **(A)** conventional but specific pathogen-free (SPF), **(B)** secondary abiotic (SAB), **(C)** murine microbiota-associated (mma), and **(D)** human microbiota-associated (hma) mice using cultural analysis and expressed as colony forming units per gram of feces (CFU/g). Box plots indicate the 25th and 75th percentiles of the median (black bar inside the box). Each dot corresponds to an individual mouse. Numbers (in parentheses) specify the number of mice included.

Cultural analysis of fecal samples from SPF mice exhibited effective clearance of the pathogen within a few days of infection, indicative for a strong CR against *C. jejuni* given a complete absence of colonization (Figure 1A). In contrast, CR was abrogated in SAB mice which revealed a robust and persistent colonization of the colon by *C. jejuni*, with pathogen densities ranging from 10^8^ to 10^9^ colony-forming units per gram (CFU/g) during the experimental period (Figure 1B). Conversely, SAB mice reconstituted with murine gut microbiota mma showed a protective CR with sporadic colonization occurring in only a few individuals (Figure 1C). On the other hand, hma mice displayed absence of CR as indicated by susceptibility to *C. jejuni* intestinal colonization, maintaining high median pathogen loads between 10^7^ and 10^9^ CFU/g of feces throughout the experiment (Figure 1D).

**Figure 2:**
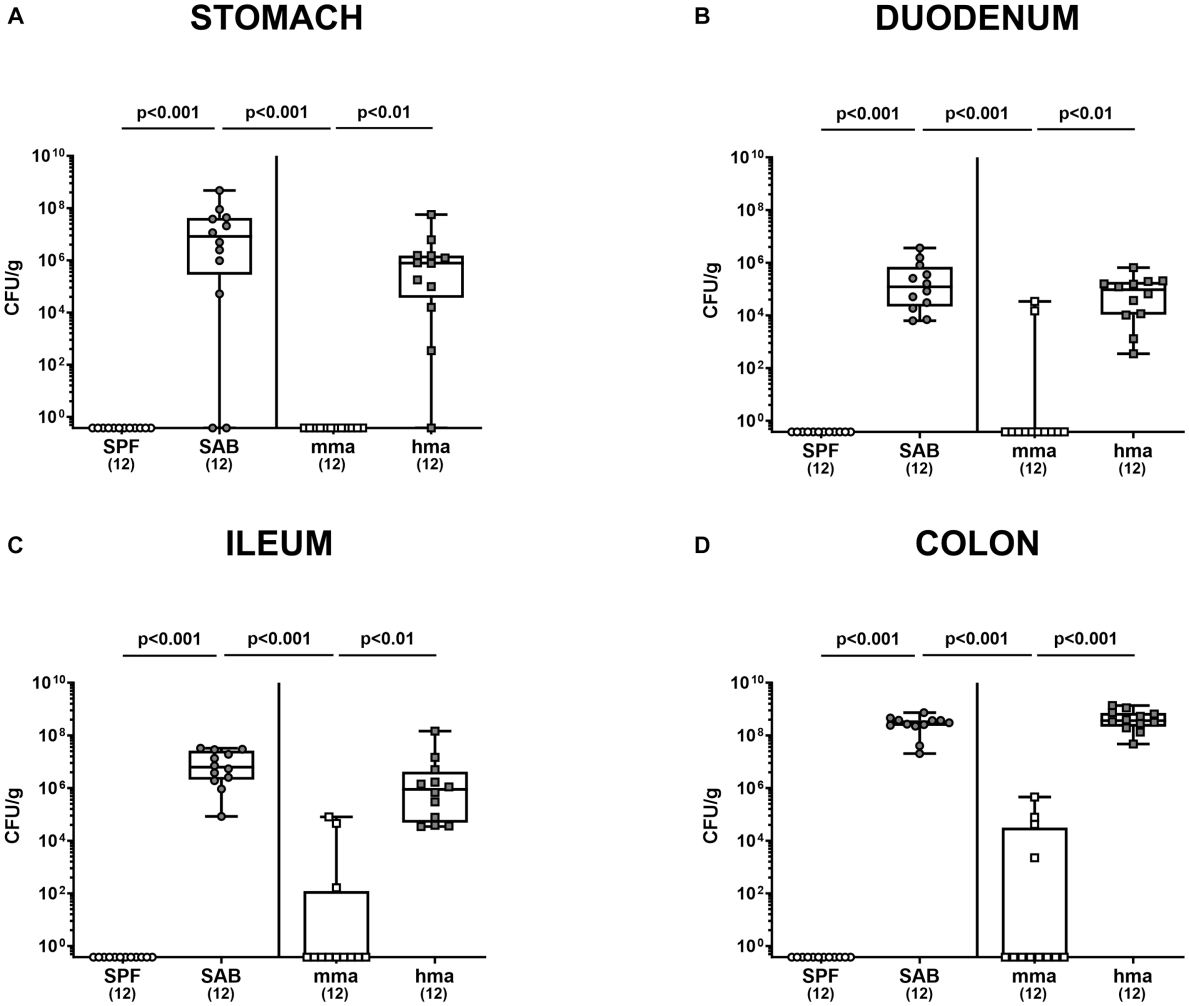
Gastrointestinal pathogen loads following *C. jejuni* infection in SPF, SAB, mma, and hma mice. Conventional but specific pathogen-free (SPF; white circles), secondary abiotic (SAB; gray circles), murine microbiota-associated (mma; white squares), and human microbiota-associated (hma; gray squares) mice were perorally infected with *C. jejuni* strain 81-176 on days 0 and 1. Cultural analysis for **(A)** stomach, **(B)** duodenum, **(C)** ileum, and **(D)** colon contents at the end of the experiment on day 21 were expressed as CFU/g of feces. Box plots indicate the 25th and 75th percentiles of the median (indicated by a black bar inside the box), as well as the range. Significance levels (*p* values) were determined by the Kruskal-Wallis test with Dunn’s post correction. Numbers (in parentheses) specify the number of mice included.

Furthermore, we investigated pathogen numbers along the gastrointestinal tract in the distinct mouse cohorts. As anticipated, CR against *C. jejuni* observed in SPF and mma mice extended throughout the gut, as demonstrated by significantly higher *C. jejuni* loads in the stomach, duodenum, ileum, and colon of SAB mice compared to SPF and mma mice, and in hma mice compared to mma mice (*p* < 0.01–0.001) (Figures 2A–D).

Hence, these results demonstrate that the two mice groups - with and without CR each – were excellently suited to investigate the metabolite patterns in the intestinal milieu associated with presence and absence of murine CR against *C. jejuni*.

### 3.2 Microbiota composition in mice with and without CR

**Figure 3:**
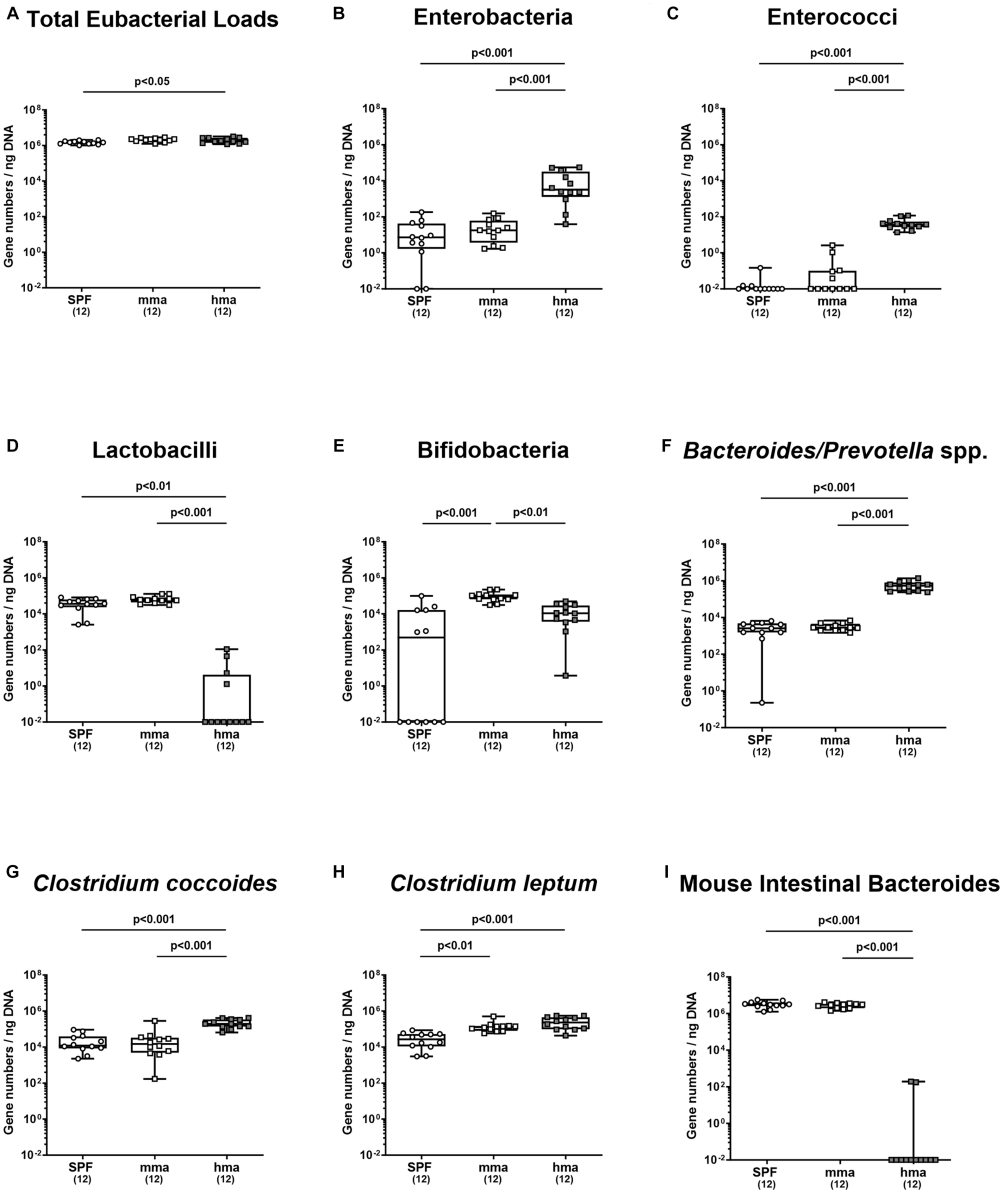
Fecal microbiota composition before and after *C. jejuni* infection. Mice were infected perorally with *C. jejuni* strain 81-176 on days 0 and 1. Gut microbiota composition was determined immediately before infection (day 0) for **(A)** total eubacterial loads, **(B)** enterobacteria, **(C)** enterococci, **(D)** lactobacilli, **(E)** bifidobacteria, **(F)** *Bacteroides/Prevotella* species, **(G)** *Clostridium coccoides* group, **(H)** *Clostridium leptum* group, and **(I)** Mouse Intestinal Bacteroides, using culture-independent, molecular methods (expressed as gene copies per ng DNA). Box plots indicate the 25th and 75th percentiles of the median (black bar inside the box). Each dot corresponds to an individual mouse. Significance levels (*p* values) are determined by the Mann–Whitney test with Tukey’s post correction or Kruskal-Wallis test with Dunn’s post correction. Numbers (in parentheses) show the number of mice included.

To further ensure the quality of our mice models with and without CR, we quantified the major microbial groups in the gut microbiota by qRT-PCR based on 16S rRNA analysis before infection. Our objective was to elucidate the differences in microbial communities between mice harboring human or murine gut microbiota known from earlier studies (Bereswill et al., 2011; Mrazek et al., 2019) to ensure that the different microbiota transplants had successfully engrafted in the mouse intestines. Our analysis revealed that hma mice harbored higher total eubacterial loads compared to SPF mice (*p* < 0.05), while no significant differences in the gene numbers were observed between SPF and mma, and between hma and mma mice (Figure 3A). Moreover, significantly higher enterobacterial loads were observed in hma mice (~10^3^ gene numbers/ng DNA) prior to infection compared to SPF and mma mice (~10 gene numbers/ng DNA) (*p* < 0.001) (Figure 3B). Differences in enterococci fecal loads were evident, with hma mice exhibiting significantly higher loads (~10^2^ gene numbers/ng DNA) than both murine models (~10^−2^ gene numbers/ng DNA) (*p* < 0.001) (Figure 3C). Similarly, in line with the distinctive species composition of the gut microbiota, hma mice harbored significantly lower fecal loads of lactobacilli (~10^−2^ gene numbers/ng DNA) compared to the resistant models, SPF and mma mice (~10^5^ gene numbers/ng DNA) (*p* < 0.01–0.001) (Figure 3D). Interestingly, mma mice displayed higher bifidobacterial loads (~10^4^ gene numbers/ng DNA) (*p* < 0.01–0.001) compared to both SPF and hma mice (~10^3^ and 10^4^ gene numbers/ng DNA, respectively) (Figure 3E). Furthermore, the levels of *Bacteroides*/*Prevotella* species were significantly elevated in hma mice (~10^6^ gene numbers/ng DNA) compared to SPF and mma mice (~10^4^ gene numbers/ng DNA) (*p* < 0.001) (Figure 3F). Similarly, hma mice also exhibited significantly higher loads (~10^5^ gene numbers/ng DNA) of the *Clostridium coccoides* group than SPF and mma mice (~10^4^ gene numbers/ng DNA) (*p* < 0.001) (Figure 3G). On the other hand, both hma and mma mice displayed higher fecal loads (~10^5^ gene numbers/ng DNA) of the *Clostridium leptum* group compared to SPF mice (~10^4^ gene numbers/ng DNA) (*p* < 0.01–0.001) (Figure 3H). Lastly, SPF and mma mice exhibited significantly higher loads (~10^6^ gene numbers/ng DNA) (*p* < 0.001) of Mouse Intestinal Bacteroides compared to hma mice, where these bacteria were nearly absent (Figure 3I). Hence, murine CR against *C. jejuni* is associated with higher loads of lactobacilli and Mouse Intestinal Bacteroides, but with lower loads of enterobacteria, enterococci, *Bacteroides/Prevotella* species, and *Clostridium coccoides* group. The fact that these results were comparable to observations in earlier studies confirm the successful establishment of the murine and human microbiota in the intestines of mice following oral FMT, and that CR was associated with a specific murine microbiota composition. Finally, species-specific differences in the microbiota compositions associated with presence and absence of CR are further used as a basis for the interpretation of results obtained from metabolomic analyses.

### 3.3 Metabolomic profiling of amino acid concentrations

**Figure 4:**
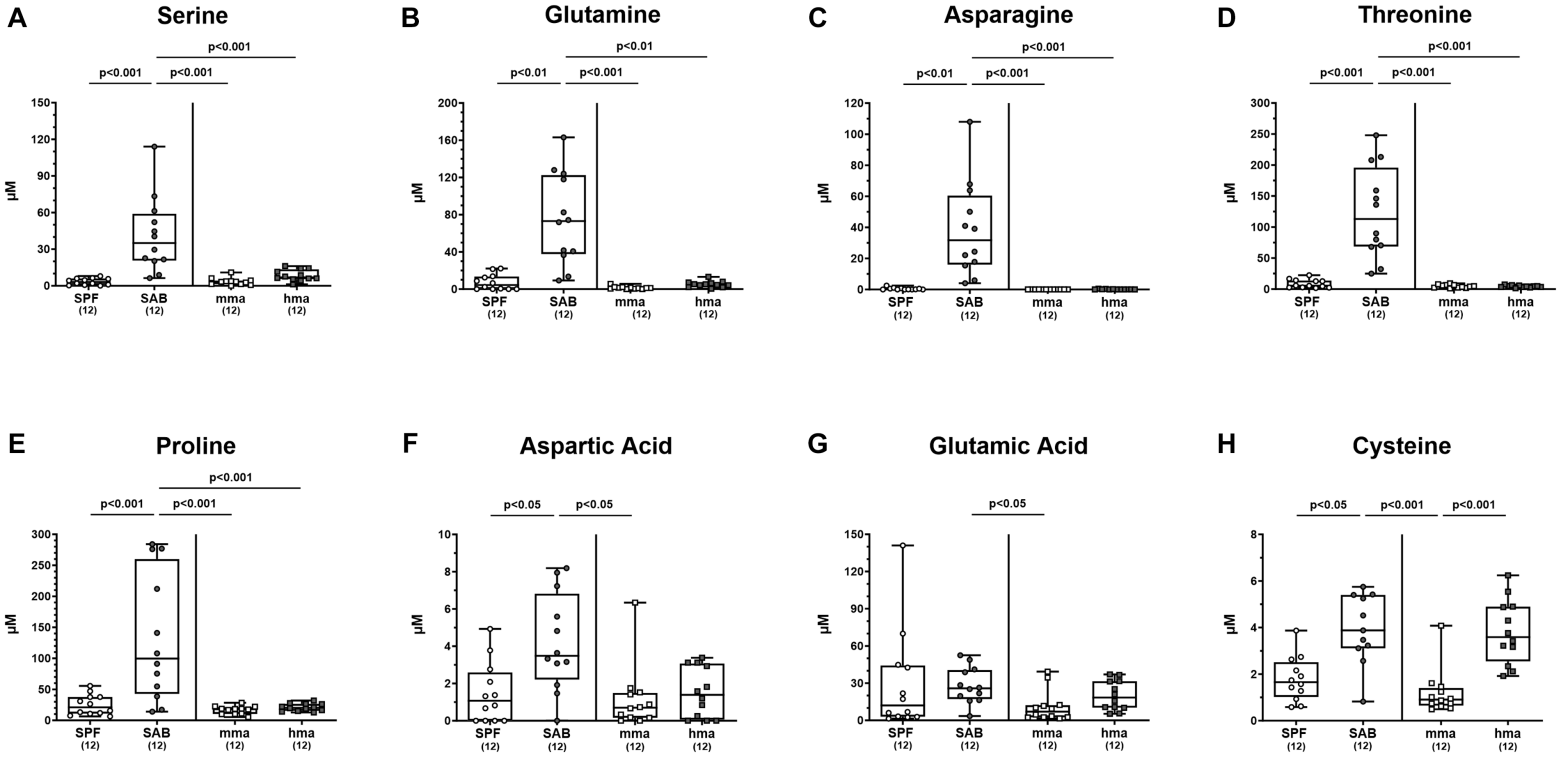
Fecal concentrations of amino acids essential for *C. jejuni* growth, in mice with and without CR. Fecal samples from conventional but specific pathogen-free (SPF) mice (*n* = 12), secondary abiotic (SAB) mice (*n* = 12), murine microbiota-associated (mma) mice (*n* = 12), and human microbiota-associated (mma) mice (*n* = 12) were harvested on day 0 before infection. Fecal levels of the amino acids **(A)** serine, **(B)** glutamine, **(C)** asparagine, **(D)** threonine, **(E)** proline, **(F)** aspartic acid, **(G)** glutamic acid and **(H)** cysteine were analyzed by LC–MS/MS, before *C. jejuni* infection. Metabolite concentration was expressed by μM. Box plots indicate the 25th and 75th percentiles of the median (black bar inside the box), as well as the total range. Significance levels (*p* values) were determined by the Mann–Whitney test with Tukey’s post correction or Kruskal-Wallis test with Dunn’s post correction, and numbers (in parentheses) indicate the number of mice included.

In consideration of *C. jejuni* relying on amino acids as a primary nutrient and energy source within the gut (Wright et al., 2009), we conducted a comprehensive analysis of the amino acid concentrations in fecal samples of mice with and without CR prior to infection. The resulting metabolomics assessment revealed notable variations in the composition of amino acids among the different cohorts (Figure 4). As could be expected due to the absence of intestinal bacteria, the SAB mice exhibited significantly higher total concentrations of free amino acids compared to the other mice groups (*p* < 0.05–0.001) (Supplementary Figure S1). Concerning individual amino acids, we focused first on the amino acids essential for *C. jejuni* growth. Most importantly, levels of cysteine which is required for *C. jejuni* growth in the absence of serine, were significantly reduced in SPF mice and in mma as compared to hma and to SAB mice (*p* < 0.05–0.001) (Figure 4H). Notably, this was the only amino acid that was significantly reduced in mma mice in comparison to hma mice. Furthermore, the abundance of other amino acids essential for fueling *C. jejuni* metabolism including serine, glutamine, asparagine, threonine, and proline, was markedly lower in SPF, mma, and hma mice compared to SAB mice (*p* < 0.01–0.001) (Figures 4A–E). On the other hand, aspartic acid concentrations were lower in SPF and mma mice compared to SAB mice (*p* < 0.05), and glutamic acid levels were lower only in mma mice compared to SAB (*p* < 0.05), nevertheless a trend toward lower concentrations of both amino acids in mma mice compared to hma mice was observed (Figures 4F,G). Similarly, this pattern extended to arginine with significantly lower concentrations in SPF mice compared to SAB mice and in mma mice compared to hma and to SAB mice (*p* < 0.05–0.001) (Supplementary Figure S2A).

Moreover, SPF mice exhibited substantially lower concentrations of other amino acids, including leucine, isoleucine, lysine, histidine, phenylalanine, tryptophan, tyrosine, and valine, as compared to SAB mice, with a trend observed toward lower concentrations in mma mice compared to hma mice (*p* < 0.05–0.001) (Supplementary Figures S2B–I). Noteworthy, the levels of these amino acids were also significantly lower in both hma and mma mice compared to SAB mice (*p* < 0.05–0.001) (Supplementary Figures S2B–I). Additionally, glycine concentrations were significantly lower in SPF and mma mice compared to SAB mice, with a trend observed toward lower levels in mma mice compared to hma mice (*p* < 0.05–0.01) (Supplementary Figure S2J). Similarly, methionine levels were significantly lower in SPF mice compared to SAB mice, and a trend toward lower concentrations in mma compared to hma mice was observed (*p* < 0.05) (Supplementary Figure S2K). On the other hand, no significant differences were detected for alanine levels between the different mouse cohorts, yet a trend toward lower concentrations in SPF mice compared to SAB mice and in mma mice compared to hma mice was observed (Supplementary Figure S2L). Furthermore, the amino acid-related metabolites sarcosine and phenylalanine betaine were also evaluated and revealed a distinct pattern. Sarcosine levels were significantly higher in SPF and mma mice compared to SAB and hma mice, respectively (*p* < 0.001 and *p* < 0.05, respectively) (Supplementary Figure S3A). Similarly, phenylalanine betaine levels were significantly elevated in SPF mice compared to SAB mice (*p* < 0.001), while mma mice exhibited higher levels compared to hma mice (*p* < 0.001) (Supplementary Figure S3B). Of note, mma mice also exhibited significantly lower levels of these metabolites compared to SAB mice (*p* < 0.01–0.001) (Supplementary Figures S3A,B). Hence, comparisons of metabolomic signatures among our mice cohorts revealed that murine CR against *C. jejuni* is significantly associated with a shortage in amino acids creating an unfavorable environment for *C. jejuni*.

### 3.4 Metabolomic profiling of bile acid concentrations

**Figure 5:**
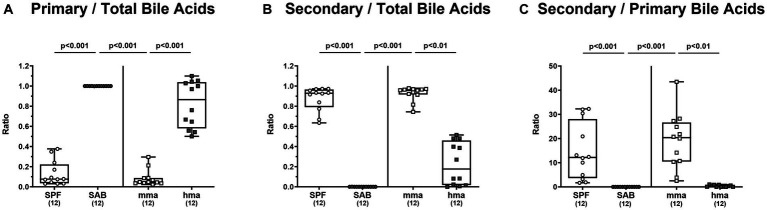
Fecal bile acid profile of mice resistant and susceptible to *C. jejuni* 81-176 infection. Fecal samples from conventional but specific pathogen-free (SPF), secondary abiotic (SAB), murine microbiota-associated (mma), and human microbiota-associated (hma) mice were harvested on day 0, and LC–MS/MS analysis was employed. The ratios of **(A)** primary bile acids concentrations as well as **(B)** secondary bile acids concentrations, to total bile acids concentrations were computed. Secondary bile acid synthesis **(C)**, defined as ratio of cytotoxic secondary bile acids to primary bile acids was also calculated. Box plots indicate the 25th and 75th percentiles of the median (black bar inside the box), as well as the total range. Significance levels (*p* values) are determined by the Kruskal-Wallis test with Dunn’s post correction. Numbers (in parentheses) show the number of mice included.

A comprehensive targeted metabolomic analysis of the bile acid composition in fecal samples of mice on d0 prior to *C. jejuni* 81-176 infection revealed significant differences between the distinct mouse cohorts. The mice with CR, namely SPF and mma mice, exhibited a bile acid profile characterized by a predominant secondary bile acid pool compared to the mice without CR, the SAB and hma mice, respectively. This was evident by a significantly lower ratio of primary to total bile acids in SPF mice compared to SAB mice, and in mma mice compared to hma and SAB mice (*p* < 0.001) (Figure 5A), as well as the significantly higher ratio of secondary to total bile acids in SPF mice compared to SAB mice, and in mma mice compared to hma and SAB mice (*p* < 0.01–0.001) (Figure 5B). Additionally, the ratio of secondary bile acids to primary bile acids, an indicator of secondary bile acid synthesis, was found to be significantly upregulated in the SPF mice compared to SAB mice (*p* < 0.001), and in mma mice compared to hma and to SAB mice (*p* < 0.01 and *p* < 0.001, respectively) (Figure 5C).

**Figure 6:**
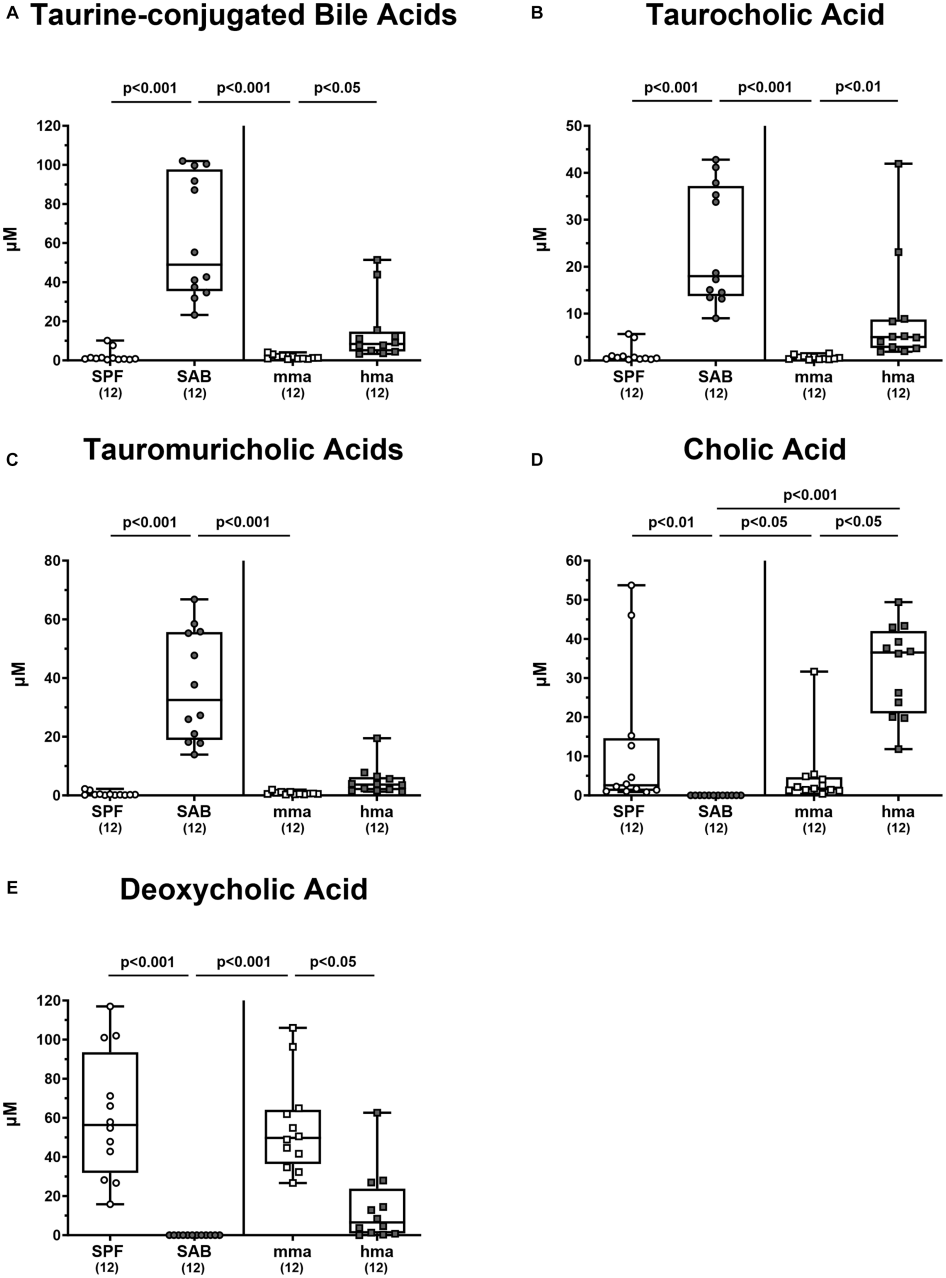
Primary and secondary bile acids in the feces of mice with and without CR. **(A)** The sum of taurine conjugated bile acids were computed. Fecal concentrations of **(B)** tauromuricholic acids, **(C)** taurocholic acid, **(D)** cholic acid, and **(E)** deoxycholic acid were measured using LC–MS/MS, from 4 mouse cohorts, SAB, SPF, hma, and mma mice on day 0 prior to infection. Metabolite concentrations are expressed in μM. Box plots reveal the 25th and 75th percentiles of the median (black bar inside the box), as well as the total range were assigned. Significance levels (*p* values) were determined by the Kruskal-Wallis test with Dunn’s post correction, and numbers (in parentheses) indicate the number of mice included.

It is of note that the secondary bile acid deoxycholic acid, which is highly toxic to *C. jejuni* was significantly associated with CR in the mice groups. It was elevated in SPF mice compared to SAB mice, as well as in mma mice compared to hma and SAB mice (*p* < 0.05–0.001) (Figure 6E).

Moreover, it was reported that tauroconjugation of cholic acid is associated with a higher bacterial 7-α-dehydroxylation into deoxycholic acid (Eldere et al., 1996). Taurine-conjugated bile acids were predominant in the bile acid pool of SAB mice and hma mice, leaving SPF mice with significantly lower levels compared to SAB mice, and mma mice with lower levels compared to hma and SAB mice (*p* < 0.05–0.001) (Figure 6A). In particular, significantly lower concentrations of taurocholic acid and tauromuricholic acids were observed in SPF and mma mice compared to SAB mice, and in mma mice compared to hma mice for taurocholic acid (*p* < 0.01–0.001) (Figures 6B,C), with a trend toward lower concentrations for tauromuricholic acids (Figure 6C). Remarkably, the primary bile acid cholic acid exhibited a distinct pattern, with SPF mice harboring significantly higher concentrations compared to SAB mice, and mma mice harboring significantly lower concentrations than hma mice (*p* < 0.05–0.01) (Figure 6D). Moreover, the cholic acid concentrations were significantly lower in SAB mice compared to mma and hma mice (*p* < 0.05 and *p* < 0.001, respectively) (Figure 6D).

Hence, the results from bile acid profiling highlight distinct metabolomic signatures associated with CR against *C. jejuni* which is characterized by a predominance of secondary bile acids.

### 3.5 Metabolomic profiling of fatty acids concentrations

**Figure 7:**
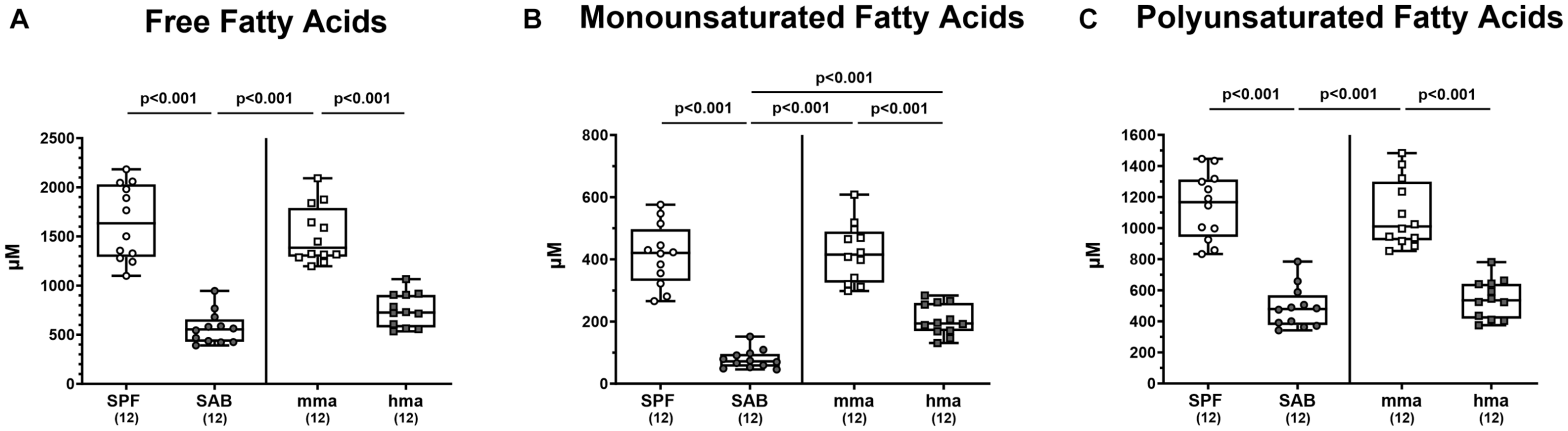
Fatty acid composition in the feces mice resistant and susceptible to *C. jejuni* infection. Fecal samples were analyzed by LC–MS/MS, from secondary abiotic (SAB) mice (*n* = 12), conventional but specific pathogen-free (SPF) mice (*n* = 12), human microbiota-associated (hma) mice (*n* = 12), and murine microbiota-associated (mma) mice (*n* = 12), to characterize the fatty acid composition in the colon of the distinct mouse cohorts on day 0 prior to infection. Sums of **(A)** free fatty acids, **(B)** monounsaturated fatty acids, and **(C)** polyunsaturated fatty acids were computed. Metabolite concentrations were expressed in μM. Box plots indicate the 25th and 75th percentiles of the median (black bar inside the box), as well as the total range. Significance levels (*p* values) were determined by one-sided ANOVA with Tukey’s post correction. The number of mice included are indicated by numbers (in parentheses).

In light of the pivotal antimicrobial roles of fatty acids and their potential impact on the composition of the gut microbiota, we conducted an in-depth investigation into the fatty acid profiles present in the colonic milieu of our distinct mouse models on day 0 before infection. Through comprehensive metabolomic analysis, we unraveled distinct patterns of fatty acid concentrations that distinguished between mouse groups exhibiting CR or susceptibility to *C. jejuni* colonization. Remarkably, our findings revealed that SPF mice exhibited significantly higher concentrations of free fatty acids in the colon compared to SAB mice (*p* < 0.001, Figure 7A). A similar trend was observed for mma mice, demonstrating significantly elevated levels of free fatty acids compared to hma and to SAB mice (*p* < 0.001, Figure 7A). Moreover, when examining the sum of monounsaturated and polyunsaturated fatty acids, SPF mice displayed significantly higher concentrations of both types of fatty acids compared to SAB mice (*p* < 0.001), while mma mice had significantly higher concentrations compared to hma and SAB mice (*p* < 0.001) (Figures 7B,C). Notably, monounsaturated fatty acid concentrations were found to be significantly higher (*p* < 0.001) in hma mice compared to SAB mice (Figure 7B).

**Figure 8:**
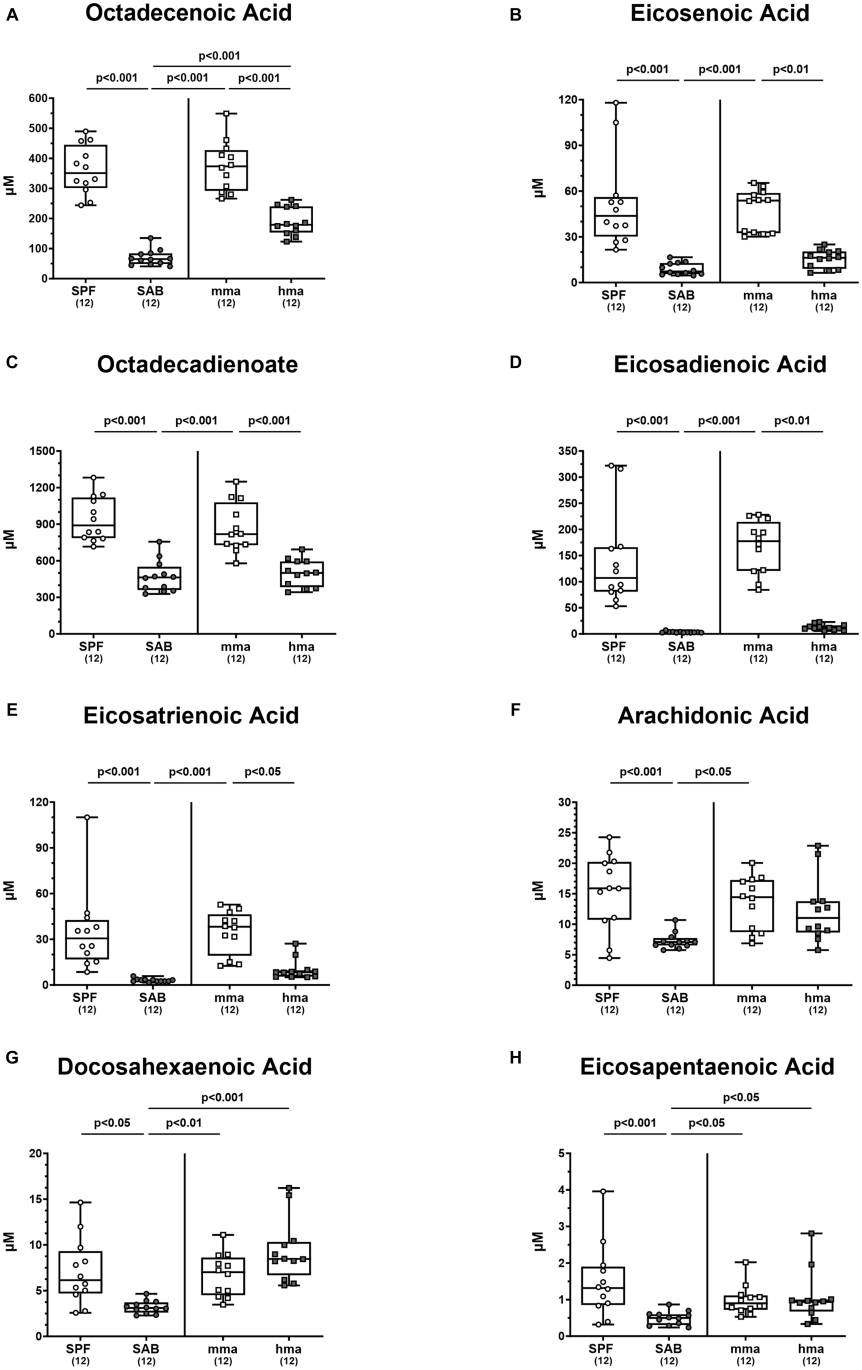
Monounsaturated and polyunsaturated fatty acids in mice with and without CR. Fecal samples were analyzed by LC–MS/MS, from conventional but specific pathogen-free (SPF), secondary abiotic (SAB), murine microbiota-associated (mma), and human microbiota-associated (mma) mice on day 0 prior to infection. The monounsaturated fatty acids **(A)** octadecenoic acid and **(B)** eicosenoic acid, as well as the polyunsaturated fatty acids **(C)** octadecadienoate, **(D)** eicosadienoic acid, **(E)** eicosatrienoic acid, **(F)** arachidonic acid, **(G)** docosahexaenoic acid, and **(H)** eicosapentaenoic acid were shown. Metabolite concentrations were expressed in μM. Box plots indicate the 25th and 75th percentiles of the median (black bar inside the box), as well as the total range. Significance levels (*p* values) were determined by one-sided ANOVA with Tukey’s post correction or the Kruskal-Wallis test with Dunn’s correction. The number of mice included are indicated by numbers (in parentheses).

Further delving into the differentially expressed fatty acids, particularly within the monounsaturated fatty acid class, we observed significant differences in octadecenoic acid and eicosenoic acid. Octadecenoic acid levels were significantly elevated in SPF and in mma mice compared to SAB and hma mice, respectively (*p* < 0.001) (Figure 8A). Additionally, hma mice displayed higher concentrations of octadecenoic acid compared to SAB mice (*p* < 0.001) (Figure 8A). A similar pattern emerged for eicosenoic acid, with SPF and mma mice exhibiting substantially elevated levels of this fatty acid compared to SAB and hma mice, respectively (*p* < 0.01–0.001) (Figure 8B), whereas no statistically significant difference was observed between the two susceptible groups (Figure 8B).

Interestingly, the distinctive patterns observed between resistant and susceptible groups extended to specific polyunsaturated fatty acids, although not all fatty acids followed these patterns. Octadecadienoate, eicosadienoic acid, and eicosatrienoic acid exhibited different patterns, with SPF mice harboring higher fecal concentrations of these fatty acids compared to susceptible models SAB mice, and mma mice harboring higher concentrations compared to hma and SAB mice (*p* < 0.05–0.001) (Figures 8C–E). On the other hand, although SPF and mma mice exhibited significantly higher fecal concentrations of arachidonic acid compared to SAB mice (*p* < 0.001 and *p* < 0.05, respectively), a trend toward higher concentrations in mma mice compared to hma mice was still observed (Figure 8F). Furthermore, SPF, mma, and hma mice displayed higher colonic concentrations of docosahexaenoic acid and eicosapentaenoic acid compared to SAB mice (*p* < 0.05–0.001) (Figures 8G,H). Nevertheless, no significant differences were observed in colonic levels of the latter fatty acids between mma and hma mice (Figures 8G,H). Hence, these results indicate that elevated free fatty acids represent another metabolomic signature significantly associated with murine CR against *C. jejuni*.

### 3.6 Other metabolites

**Figure 9:**
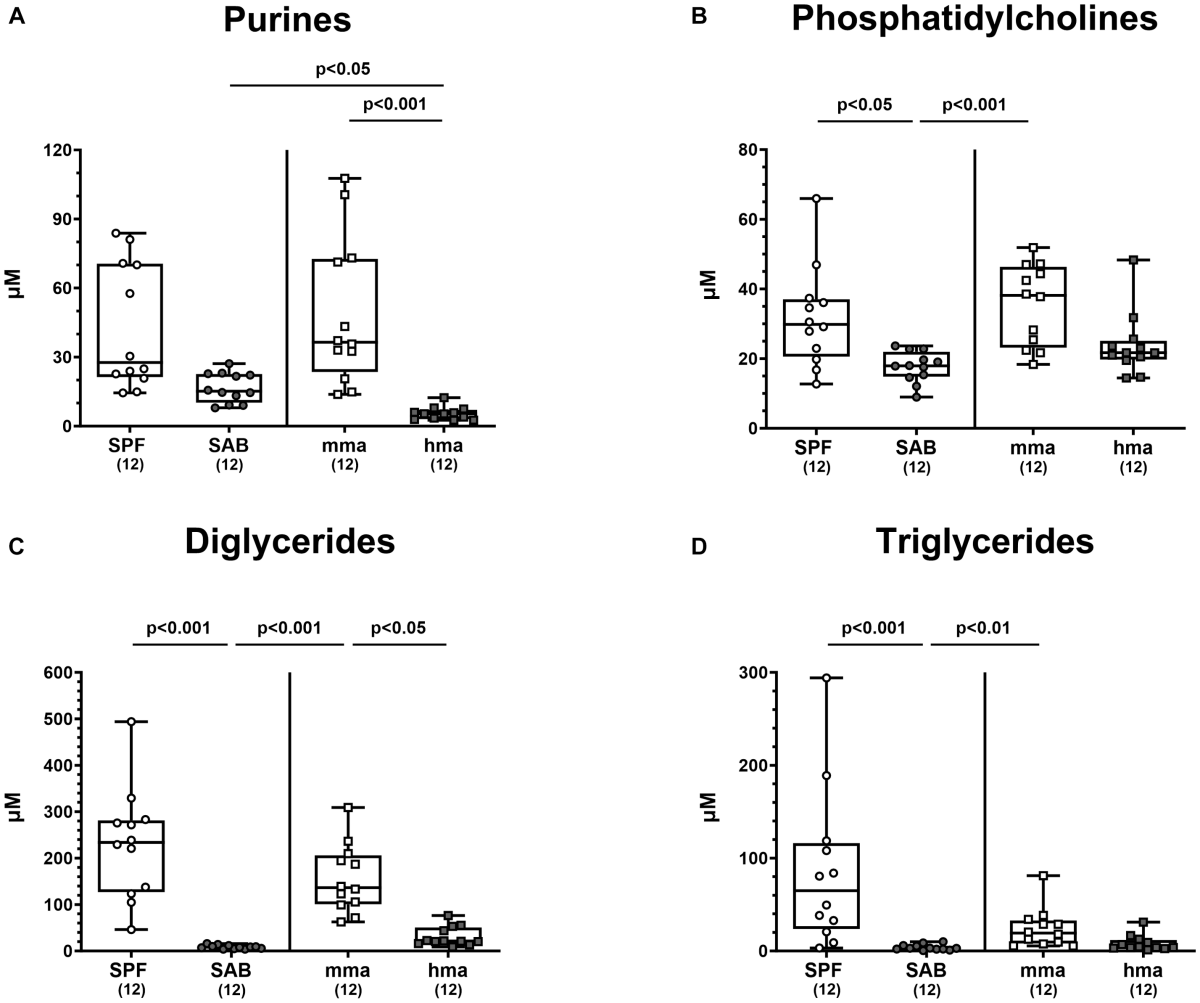
Other classes of metabolites differentially expressed between mice cohorts resistant and susceptible to *C. jejuni* infection. Fecal samples analyzed by LC–MS/MS, derived from the mice cohorts (conventional but specific pathogen-free (SPF), secondary abiotic (SAB), murine microbiota-associated (mma), and human microbiota-associated (mma) mice) on day 0 prior to infection. The sum **(A)** of purines, **(B)** phosphatidylcholines, **(C)** diglycerides, and **(D)** triglycerides were shown. Metabolite concentrations were expressed in μM. Box plots indicate the 25th and 75th percentiles of the median (black bar inside the box), as well as the total range. Significance levels (*p* values) were determined by the Kruskal-Wallis test with Dunn’s post correction. The number of mice included are indicated by numbers (in parentheses).

In addition to investigating the role of bile acids, amino acids, and fatty acids, we explored the metabolomic landscape of other classes of metabolites present in the colon of our mouse models before infection. Specifically, we delved into the analysis of phosphatidylcholines and diglycerides, which hold potential relevance to host-microbe interactions, in addition to the crucial components of nucleic acids, namely purines. Our investigation into purine metabolites revealed distinct profiles that distinguished between mice cohorts with and without CR (Figure 9A). Mma mice exhibited significantly higher levels of purine metabolites compared to hma mice (*p* < 0.001) (Figure 9A). Interestingly, although no significant differences were observed between SAB and the resistant cohorts, a trend was still observed, and SAB mice displayed higher concentrations of purines compared to hma mice (*p* < 0.05) (Figure 9A). In particular, xanthine concentrations in SPF, mma, and SAB mice were significantly higher compared to hma mice (*p* < 0.01–0.001) (Supplementary Figure S4A). On the other hand, hypoxanthine displayed a different pattern, as SPF mice harbored significantly higher levels compared to SAB mice, and this was also the case for mma mice compared to hma and SAB mice (*p* < 0.01–0.001) (Supplementary Figure S4B). Notably, SAB mice exhibited a higher xanthine synthesis computed by the ratio of xanthine to hypoxanthine compared to both SPF and mma mice (*p* < 0.001) (Supplementary Figure S4C).

Moreover, these distinct patterns extended to phosphatidylcholines, diglycerides, and triglycerides. SPF and mma mice exhibited significantly higher concentrations of all three classes of the metabolites compared to SAB mice (*p* < 0.05–0.001) (Figures 9B–D). Nevertheless, compared to hma mice, only diglycerides were measured at significantly higher concentrations in mma mice (*p* < 0.05), albeit a trend toward higher levels of phosphatidylcholines and triglycerides in mma mice compared to hma mice could still be observed (Figures 9B–D).

## 4 Discussion

Murine models have proven invaluable in uncovering the complex interplay between host, microbiota, and intestinal pathogens. The intricate role of the gut microbiota in murine CR against *C. jejuni* represents a pivotal step in disease initiation, progression, and pathogenesis of campylobacteriosis. Comparing SPF and microbiota-depleted (i.e., SAB) mice reveals metabolomic signatures associated with the presence or absence of the gut microbiota. Additionally, contrasting hma mice with mma or SPF counterparts unveils species-specific metabolic profiles. In order to identify metabolites of potential benefit for prevention and treatment of *C. jejuni* infection, we employed a comprehensive metabolomics profiling approach to shed light on the metabolome signatures and distinct metabolites associated with CR against the gut pathogen.

Our findings demonstrate robust CR against *C. jejuni* 81-176 in the colon of SPF mice which is abrogated by antibiotic treatment in SAB mice, confirming the crucial role of the gut microbiota in CR and in preventing pathogen growth as shown earlier (Bereswill et al., 2011) and confirmed here (Figures 1, 2). In addition, the restoration of CR in mma mice but not in hma mice nicely proved that the characteristic species-specific microbiota was established by FMT of SAB mice in mma as well as hma mice and reproduce again our data concerning the specific host’s endogenous gut microbiota composition in previous studies (Bereswill et al., 2011; Ducarmon et al., 2019; Mousavi et al., 2021; Herzog et al., 2023; Shayya et al., 2023). Notably, our findings suggest that lactobacilli are associated with CR against *C. jejuni*, as supported by the notable increase in lactobacilli levels observed in SPF and mma mice with CR, as compared to the hma mice without CR (Figure 3), which was also true for Mouse Intestinal Bacteroides. These results align with previous studies demonstrating the beneficial effects of lactobacilli in inhibiting various other enteric pathogens, including *Salmonella* (Bernet-Camard et al., 1997), *E. coli* (Mangell et al., 2002), and *Listeria monocytogenes* (de Waard et al., 2002). Importantly, some studies have also reported a protective effect of lactobacilli in preventing *Campylobacter* infections (Morishita et al., 1997; Willis and Reid, 2008; Wagner et al., 2009). However, monocolonization with a single *Lactobacillus johnsonii* strain isolated from our mice was not able to protect SAB mice from *C. jejuni* infection, indicating that the role of individual *Lactobacillus* species in CR is complex and needs to be analyzed in more detail in ongoing studies (Bereswill et al., 2017).

Interestingly, treating human intestinal epithelial cells with *Lactobacillus helveticus* R0052 reduced *C. jejuni* invasion into these cells, suggesting a contribution of competitive exclusion by adherent bacteria (Wine et al., 2009). Another study also reported the capacity of other *Lactobacillus* strains to prevent *C. jejuni* adhesion and invasion to intestinal cells (Wang et al., 2014). Finally, the lactobacilli dominating in the murine intestinal tract might inhibit *C. jejuni* growth by lowering the pH and by production of bacteriocins as shown earlier (Robyn et al., 2012; Zommiti et al., 2016; Kobierecka et al., 2017).

In addition, nutrient competition is a key factor in driving CR against bacterial pathogens (Maltby et al., 2013). Thus, the antimicrobial effects of lactobacilli mentioned above might be further supported by consumption of amino acids leading to out-competition of *C. jejuni*. In contrast to other gut bacteria, the lactobacilli colonize the entire gastrointestinal tract including upper parts like the forestomach and small intestine at high numbers. In consequence, consumption by lactobacilli might add substantially to the scarcity of essential amino acids for *C. jejuni* established in mice with CR. This is supported by significantly higher levels of free amino acids detected in SAB mice as compared to the other mouse cohorts (Supplementary Figure S1) which can be attributed to the absence of a complex gut microbiota that typically metabolize and utilize these nutrients. Without microbial nutrient competition, these amino acids are utilized by *C. jejuni* for the multiplication required for gut colonization. *C. jejuni* utilize transporters and other uptake systems to acquire essential amino acids, including cysteine, serine, aspartate, asparagine, glutamate, glutamine, threonine, and proline (Velayudhan et al., 2004; Hofreuter et al., 2006; Leon-Kempis Mdel et al., 2006; Heimesaat et al., 2023). Most importantly, cysteine has been shown to be essential for *C. jejuni* 81-176 growth *in vitro* (Vorwerk et al., 2014; Hitchcock et al., 2022). To overcome this limitation, *C. jejuni* utilize free cysteine and cysteine-containing peptides to fuel respiration as a sulfur source. Unlike other enteropathogenic bacteria, *C. jejuni* cannot obtain cysteine from sulfur (Vegge et al., 2009; Man et al., 2020) and thus, cysteine acts as a chemoattractant for *C. jejuni*, and is required for the vitality of this pathogen. Our data clearly highlight significantly elevated levels of free cysteine available in the colonic environment of mice without CR, namely SAB and hma mice (Figure 4), indicating that the scarcity of cysteine in SPF and mma mice might support the CR in these murine infection models. In this context it is noteworthy that some *Lactobacillus* species depend on cysteine uptake from the environment (Bloom et al., 2022). Thus, lactobacilli might compete for cysteine with *C. jejuni*.

Interestingly, elevated levels of the other essential amino acids were observed in SAB mice (Figure 4). This, however, was not observed in hma mice, which displayed different patterns in amino acid levels.

Our metabolome analyses revealed that concentrations of bactericidal molecules including bile acids and fatty acids were elevated in fecal samples derived from mice with CR as compared to mice without CR. This points toward a direct killing of *C. jejuni* in the colonic milieu of SPF and mma mice with a murine gut microbiota. Both molecular classes may represent key drivers of CR against the pathogen. Bile acids are synthesized in the liver and secreted into the intestinal tract to aid in digesting dietary lipids. Conjugation with glycine or taurine increases their solubility and this conjugation can be modified by gut bacteria (Hofmann, 1999). Consistent with our findings, SAB mice, which lack microbial bile acid transformations, exhibited an exclusive composition of liver-derived tauro-conjugated primary bile acids (Figure 6). On that note, tauroconjugated cholic acid enhances bacterial 7-α-dehydroxylation into deoxycholic acid, essentially by providing a sulfur source for the anabolic pathways involved in dihydroxylation (Eldere et al., 1996). Interestingly, our findings indicate a depletion of tauroconjugated bile acids in the colonic environment of mice with CR as compared to the susceptible cohorts (Figure 6), suggesting their potential transformation to secondary bile acids, which were elevated in the resistant mice compared to the susceptible ones (Figure 5). The majority of conjugated bile acids are reabsorbed in the distal ileum, while the remaining undergo bacterial metabolism in the colon, particularly through 7α-dehydroxylation carried out by a few bacteria, primarily belonging to the *Clostridium* species, resulting in the production of secondary bile acids such as deoxycholic acid which exerts a strong antimicrobial activity (Hirano et al., 1981). Importantly, bacterial deconjugation of conjugated bile acids by bile salt hydrolase (BSH), which is highly expressed in lactobacilli, represents a key regulator in bile acid metabolism into secondary bile acids (Jia et al., 2019). This is well in line with the elevated loads of lactobacilli in SPF and mma mice with CR as compared to the susceptible hma mice (Figure 3D). Additionally, BSH activity profoundly influences host lipid metabolism, linking it to fecal fatty acids levels in our murine models (Joyce et al., 2014). We suggest that the robust BSH activity in SPF mice, and similarly in mma mice, indicated by diminished taurine-conjugated bile acids, leads to a reduction in fatty acid resorption, increasing the fecal fatty acid content. Conversely, SAB mice exhibit elevated taurocholic acids, which increases fatty acid resorption, thus depleting fecal fatty acids. On the other hand, hma mice exhibit an intermediate phenotype, with moderate BSH activity leading to a moderate fatty acid resorption. Deoxycholic acid has been shown to exhibit potent bactericidal effects against *C. jejuni* (Kurdi et al., 2006; Sannasiddappa et al., 2017; Thanissery et al., 2017). Previous studies have highlighted the critical role of the CmeABC efflux pump in *C. jejuni*’s resistance to bile acids and subsequent intestinal colonization (Lin et al., 2003). Moreover, it has been shown that *C. jejuni* accommodates a well-developed chemotaxis system that repels the pathogen from bile constituents in particular secondary bile acids (Hugdahl et al., 1988; Vegge et al., 2009). The MIC and MIC50 values of deoxycholic acid against *C. jejuni* strain 81-176 (determined in our laboratories as described in methods) were 256 mg/L and 128 mg/L, respectively. Therefore, it is not surprising that deoxycholic acid effectively reduced *C. jejuni* 81-176 colonization of the intestinal tract of broiler chickens (Alrubaye et al., 2019). The concentration of deoxycholic acid measured by targeted metabolomics in murine fecal matter was 94 mg/L, high enough to exert antimicrobial effects against *C. jejuni*. However, treatment of *C. jejuni* 81-176 infection of germ-free IL-10^−/−^ mice with deoxycholic acid below the MIC did not decrease densities but ameliorated some disease manifestations (Sun et al., 2018).

In addition, fecal samples of mice with CR contained elevated levels of fatty acids including medium-chain fatty acids (MCFAs) such as octadecenoic acid, an isomer of oleic acid, as compared to mice without CR (Figure 8). Long-chain fatty acids exerting potent antimicrobial properties play a crucial role in modulating the microbiota composition by selecting for resistant gut bacterial species (Alcock and Lin, 2015). It has been demonstrated that oleic acid effectively eradicates *Campylobacter* on chicken skins (Hinton and Ingram, 2000), and *in vitro* analyses revealed a potent bactericidal effect of MCFAs and long-chain fatty acids against *C. jejuni* (Sprong et al., 2001). Further studies reported that feeding broiler chicks with different concentrations of MCFAs diet led to less distinct pathogen colonization along the gastrointestinal tract (Van Deun et al., 2008; de los Santos et al., 2009, 2010; Metcalf et al., 2011). In addition, our results clearly indicate that high concentrations of diglycerides and triglycerides are associated with CR against *C. jejuni* in mice (Figures 9C,D). Diglycerides derived from fatty acids display potent antimicrobial activities. Even though triglycerides were not described to have potent antimicrobial activities, nevertheless, upon hydrolysis, these lipids release free fatty acids and monoglycerides, which in turn possess potent antimicrobial properties (Churchward et al., 2018; Yoon et al., 2018; Fischer, 2020).

Another metabolite of interest is hypoxanthine, which was found to be significantly associated with CR in our mice models. While there is no cumulative evidence to suggest that hypoxanthine itself possesses direct antibacterial properties, it can be metabolized into other compounds such as xanthine and uric acid, that have been shown to exhibit antimicrobial features (Martin et al., 2004). According to our data, CR mice displayed higher levels of xanthine compared to hma mice (Supplementary Figure S4), although SAB mice also harbored high xanthine concentrations.

Finally, these findings highlight the fact that murine CR against *C. jejuni* is characterized by complex interactions between the gut microbiota, amino acids, bile acids, and lipids. It is noteworthy that the congruence between the metabolite concentrations in our hma mice and those reported in human fecal samples, utilizing the same analytical kit (Erben et al., 2021), underscores the translational relevance of our findings. This further validates the hma mouse model as a representation of human metabolomic profiles. It is clear that CR against *C. jejuni* cannot be solely attributed to a single metabolite. Thus, the precise role of the metabolites identified here in *C. jejuni* clearance has yet to be determined. Nevertheless, based on the results presented, it is plausible that antimicrobial activities act together with substrate competition to support murine CR against *C. jejuni*.

## 5 Conclusion

Investigations into murine CR against *C. jejuni* using a comprehensive metabolomic profiling approach shed light on the dynamic gut microbiota interactions of *C. jejuni* and uncover distinct metabolome signatures associated with CR against this enteropathogen. The findings that concentrations of bactericidal molecules including secondary bile acids, fatty acids, and di-glycerides were elevated in mice with CR as compared to mice without CR points toward a growth inhibition or direct killing of *C. jejuni* in SPF and mma mice, indicating that respective metabolites may represent key drivers of CR against the pathogen. Notably, the scarcity of amino acids, which are essential for fueling *C. jejuni* growth, in mice with CR provide evidence that antimicrobial effects might be supported by out-competition mediated by lactobacilli or other gut bacterial species via similar substrate requirements. Importantly, these murine models provide valuable insights into the dynamic relationship between the vertebrate host, microbiota, and pathogen, offering a deeper understanding of metabolite-driven processes underlying CR. In addition to its implications in understanding colonization resistance against *C. jejuni*, the alignment between the hma mouse model and human metabolites enhances our comprehension of host-microbiota interaction and positions the hma mice as a versatile model for investigating a spectrum of microbiota-related metabolic phenomena (Erben et al., 2021). However, the complexity of the gut microbiota and the multitude of metabolic interactions therein necessitate further studies to decipher the specific mechanisms by which distinct metabolites and metabolic pathways influence different physiological functions, and particularly colonization resistance against *C. jejuni* in the intestinal milieu. Additionally, we employed a targeted approach for the metabolomics and microbiota composition analyses. For that, using untargeted approaches could offer a more comprehensive view of gut microbiota composition and of the metabolites, thus extending our understanding of host-microbiota interplay. Respective findings will pave the way for the broader goal of using mechanisms of CR for developing novel therapeutic approaches to combat and prevent campylobacteriosis and possibly other enteric infections.

## Data availability statement

The raw data supporting the conclusions of this article will be made available by the authors, without undue reservation.

## Ethics statement

The animal study was approved by Landesamt für Gesundheit und Soziales, Berlin. The study was conducted in accordance with the local legislation and institutional requirements.

## Author contributions

NS: Investigation, Formal analysis, Visualization, Writing – original draft. RB: Investigation, Writing – review & editing. LB: Investigation, Writing – review & editing. SM: Investigation, Writing – review & editing. SB: Writing – review & editing, Conceptualization, Funding acquisition, Supervision, Validation. MH: Conceptualization, Funding acquisition, Supervision, Validation, Writing – review & editing, Investigation.
