# Supplementary material for: Metabolomic signatures of intestinal colonization resistance against Campylobacter jejuni in mice
Source: Front Microbiol. 2023 Dec 18;14:1331114. doi: 10.3389/fmicb.2023.1331114 (PMC10757985; doi:10.3389/fmicb.2023.1331114)
Supplement: Supplementary file 1 [file Data_Sheet_1.PDF]

frontiers

Supplementary Material

Metabolomic Signatures of Intestinal Colonization Resistance against *Campylobacter jejuni* in Mice

Nizar W. Shayya [1], Rasmus Bandick[1], Lia Busmann[1], Soraya Mousavi[1], Stefan Bereswill[1#] and Markus M. Heimesaat[1#*]

*** Correspondence:** Markus M. Heimesaat: markus.heimesaat@charite.de

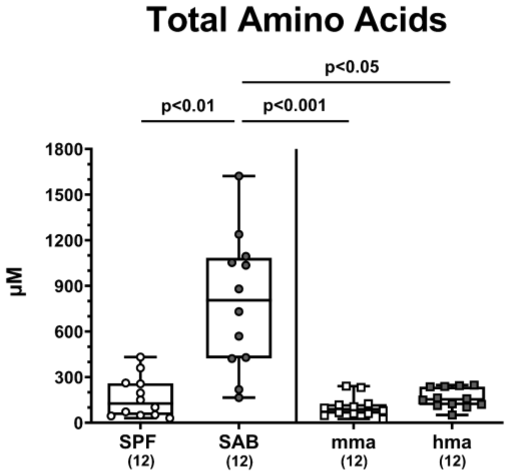

Supplementary Figure 1. Sum of free amino acids in the feces of mice with and without CR. Fecal samples from SPF mice (n=12), SAB mice (n=12), mma mice (n=12), and hma mice (n=12) were harvested and amino acids were analyzed by LC-MS/MS, before *C. jejuni* infection. Metabolite concentration was expressed by µM. Box plots indicate the 25th and 75th percentiles of the median (black bar inside the box), as well as the total range. Significance levels (p values) were determined by the Kruskal-Wallis test with Dunn's post correction. Numbers (in parentheses) indicate the number of mice included.

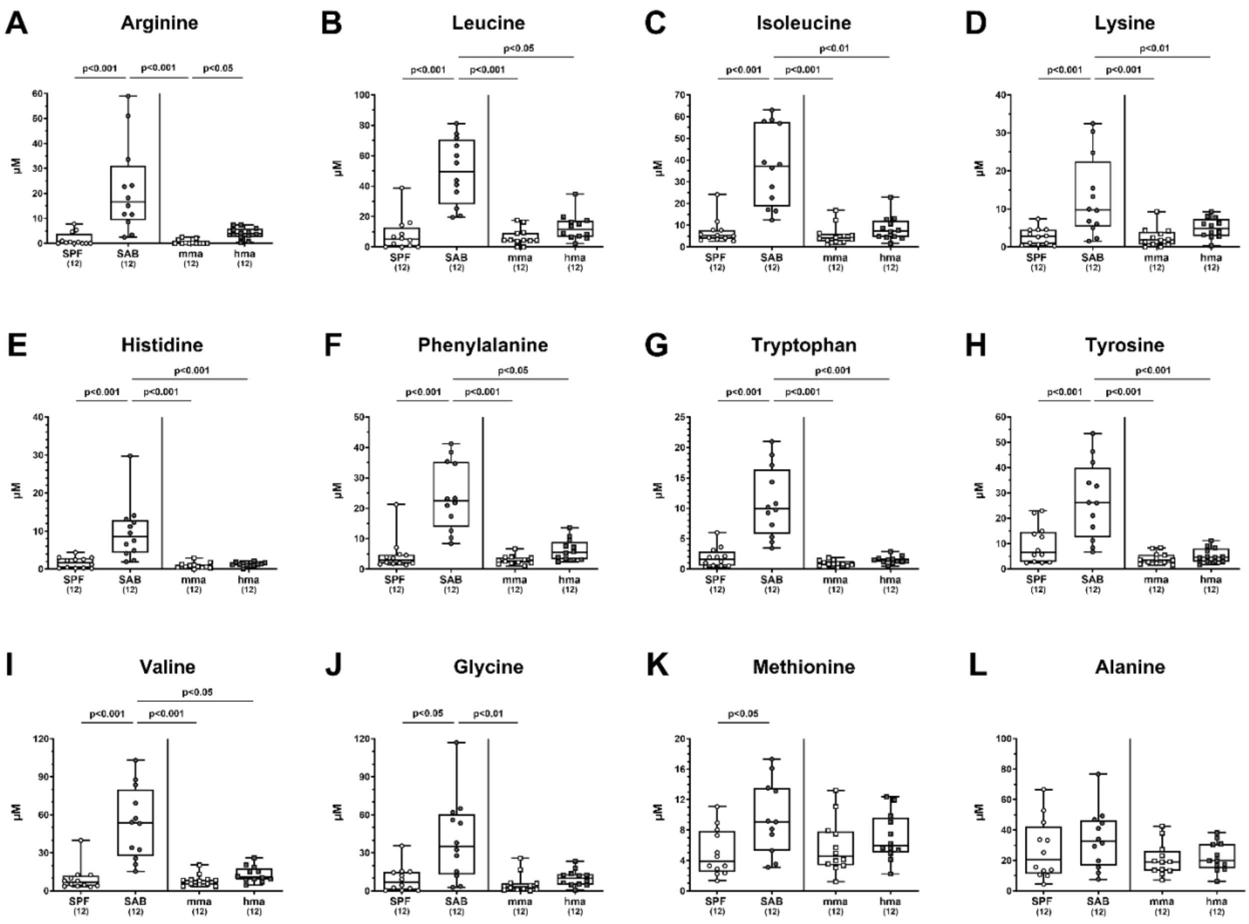

Supplementary Figure 2. Fecal concentrations of other amino acids in mice with and without CR. Fecal samples from SPF mice (n=12), SAB mice (n=12), mma mice (n=12), and hma mice (n=12) were collected. Amino acids **(A)** arginine, **(B)** leucine, **(C)** isoleucine, **(D)** lysine, **(E)** histidine, **(F)** phenylalanine, **(G)** tryptophan, **(H)** tyrosine, **(I)** valine, **(J)** glycine, **(K)** methionine and **(L)** alanine were analyzed by LC-MS/MS, before *C. jejuni* infection. Metabolite concentration was expressed by µM. Box plots indicate the 25th and 75th percentiles of the median (black bar inside the box), as well as the total range. Significance levels (p values) were determined by the Kruskal-Wallis test with Dunn's post correction for non-normally distributed data or one-sided ANOVA with Tukey correction for normally distributed data. Numbers (in parentheses) indicate the number of mice included.

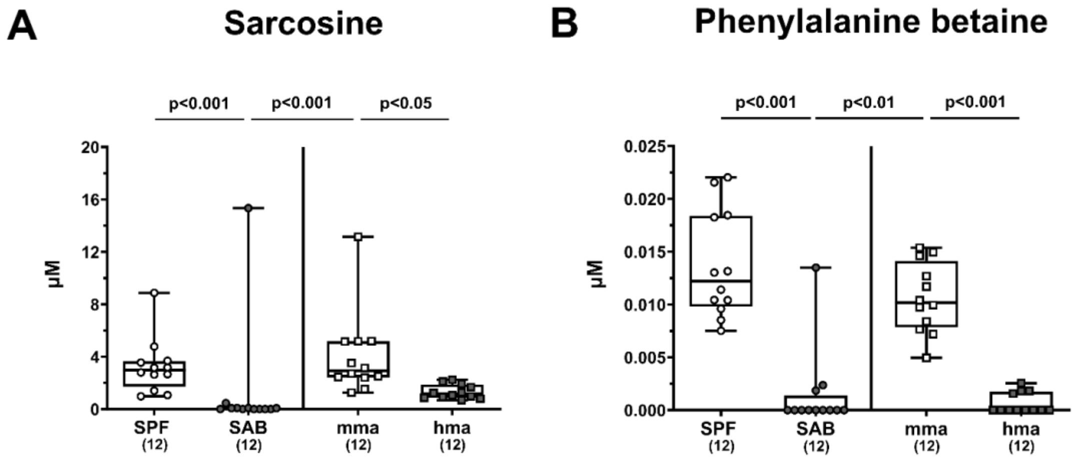

Supplementary Figure 3. Fecal concentrations of amino acid-related metabolites in mice with and without CR. Fecal samples from SPF mice (n=12), SAB mice (n=12), mma mice (n=12), and hma mice (n=12) were collected. The metabolites **(A)** sarcosine and **(B)** phenylalanine betaine were analyzed by LC-MS/MS, before *C. jejuni* infection. Metabolite concentration was expressed by µM. Box plots indicate the 25th and 75th percentiles of the median (black bar inside the box), as well as the total range. Significance levels (p values) were determined by the Kruskal-Wallis test with Dunn's post correction. Numbers (in parentheses) indicate the number of mice included.

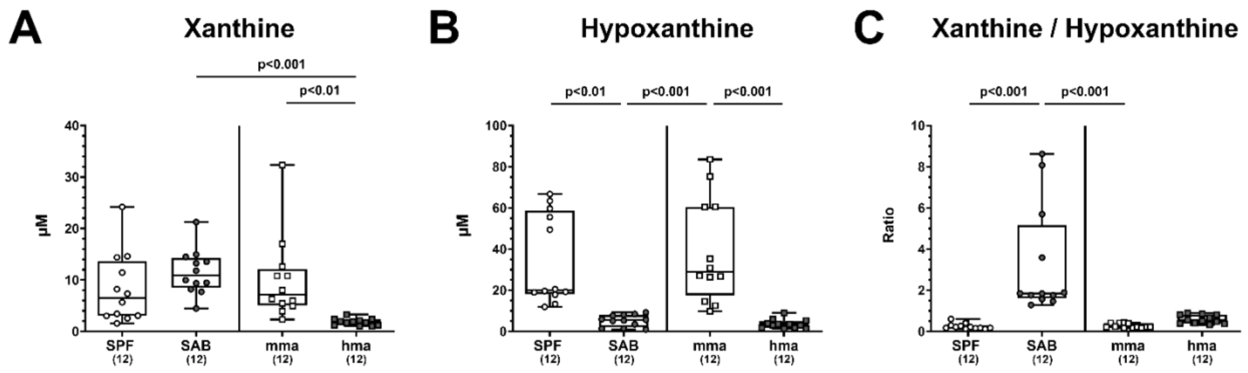

Supplementary Figure 4. Fecal concentrations of purine metabolites in mice with and without CR. Fecal samples from SPF mice (n=12), SAB mice (n=12), mma mice (n=12), and hma mice (n=12) were collected. The metabolites **(A)** xanthine and **(B)** hypoxanthine were analyzed by LC-MS/MS, before *C. jejuni* infection. **(C)** the ratio of xanthine/hypoxanthine, an indicator of xanthine synthesis was computed. Metabolite concentration was expressed by µM. Box plots indicate the 25th and 75th percentiles of the median (black bar inside the box), as well as the total range. Significance levels (p values) were determined by the Kruskal-Wallis test with Dunn's post correction. Numbers (in parentheses) indicate the number of mice included.
